# A 14 μHz/√Hz resolution and 32 μHz bias instability MEMS quartz resonant accelerometer with a low-noise oscillating readout circuit

**DOI:** 10.1038/s41378-024-00849-4

**Published:** 2024-12-23

**Authors:** Kai Bu, Cun Li, Hong Xue, Bo Li, Yulong Zhao

**Affiliations:** 1 State Key Laboratory for Manufacturing Systems Engineering, Xi’an Jiaotong University, 710049 Xi’an, China; 2 School of Mechanical Engineering, Xi’an Jiaotong University, 710049 Xi’an, China

**Keywords:** Electrical and electronic engineering, Sensors

## Abstract

A differential microelectromechanical system (MEMS) quartz resonant accelerometer with a novel oscillating readout circuit is proposed. The phase noise in a piezoelectric quartz resonant accelerometer has been systematically investigated. A high-performance front-end is used to extract the motional charge from a piezoelectric quartz resonator for the first time. This topology eliminates the tradeoff between the gain, bandwidth, and noise of the traditional front-end. The proposed bandpass front-end provides a 14.5 M gain at the oscillation frequency with a phase drift of 0.04°, ensuring a high-quality factor for the quartz resonator. The proposed bandpass front end also achieves input-referred current noise as low as 30.5 fA/√Hz, which helps improve the bias instability and resolution of the accelerometer. An anti-aliasing phase shifter is designed to regulate the loop bandwidth and compensate for additional phase drifts. To reduce the flicker noise introduced by the nonlinear effect, an amplitude limiter is used to set the resonator operating point. The accelerometer achieves a frequency resolution of 14 μHz/√Hz and bias instability of 32 μHz with a ± 70 g full scale, 54.5 Hz/g scale factor, and 552 Hz bandwidth.

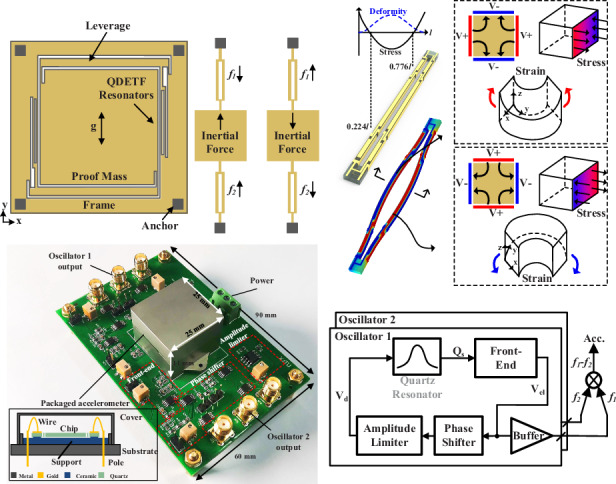

## Introduction

Microelectromechanical system (MEMS) accelerometers have a wide spectrum of applications in fields such as inertial guidance, seismic detection, wearable devices, and intelligent robots. High-resolution and low-drift acceleration measurements are among the most critical performance metrics for applications such as satellite control and unmanned underwater vehicles, which require the detection of extremely weak acceleration signals. MEMS resonant accelerometers can modulate the input acceleration signal to the carrier frequency and output the resonant frequency of the sensitive element as the measured acceleration, which can obtain a lower noise level than amplitude output accelerometers (e.g., MEMS capacitance accelerometers)^1,2^. These methods also have the advantages of high resolution, wide full scale, large dynamic range, and good environmental adaptability and have gradually become the research hotspot of high-resolution MEMS accelerometers^3,4^.

Fluctuations in the acceleration output under static input conditions (typically, a 0 g loading) can be attributed to environmental factors such as temperature field swings or background noise. Temperature-introduced trends can be compensated in several ways^5–7^, such as polynomial fitting, filtering techniques, and structural optimization. However, compensating for the white noise introduced by the Brownian motion of the resonator and electronic background using such methods is difficult, which becomes a key constraint on the accelerometer resolution.

High-resolution MEMS resonant accelerometers fabricated on silicon wafers with large-scale factors can significantly reduce acceleration white noise, which has been the focus of considerable research recently^8,9^ because of their small size, low cost, and compatibility with the semiconductor process. However, the silicon material itself has no electromechanical conversion effect and requires an additional structure to drive and detect the resonator. The typical electrostatic comb changes the dynamic behavior of the resonator, and the electrostatic force also introduces large nonlinearities and noise, limiting the linear range and resolution of the MEMS silicon resonant accelerometers^10,11^.

To obtain an ideal MEMS resonator, a material that is convenient to drive and detect is urgently needed. Monocrystalline quartz has a natural piezoelectric effect and can be driven into a specific mode by placing electrodes on its surface and applying an alternating voltage, which allows efficient conversion of electromechanical energy without additional structures. MEMS quartz resonators are compact, exhibit a high-quality factor (Q factor), and encompass a wide linear operating range^12^. Their stable crystal structure renders them optimal for the fabrication of MEMS resonant accelerometers, which offer high resolution and excellent long-term stability^13^. Recently, MEMS quartz resonant accelerometers have received widespread attention owing to their excellent performance prospects, and few studies of high-resolution MEMS quartz resonant accelerometers have been reported. Previous studies^14–17^ have revealed a method to increase the resolution by increasing the scale factor of the MEMS quartz resonant accelerometer. Although the simplicity of this method is remarkable, it also reduces the measurement range and mechanical bandwidth of the accelerometers, limiting the range of applications. An alternative approach is to increase the deformation of the resonator to improve the signal-to-noise ratio (SNR). Levy R. employed this method for seismic detection, achieving a resolution of 50 μg^18^. Nevertheless, the nonlinear effect of the quartz resonator exacerbates the bias instability of the accelerometer. The oscillating readout circuit, a key component of the MEMS quartz resonant accelerometer, provides gain and phase compensation to the quartz resonator such that it is constantly in operating mode; its accuracy and noise level significantly affect the acceleration output white noise^19^. With respect to oscillating readout circuits based on the electrostatic principle, which has been the subject of extensive research^9,10^, the study of oscillating readout circuits for quartz resonant accelerometers faces new challenges. These challenges include vibration mode driving and electrode design based on the inverse piezoelectric effect, high-resolution extraction of dynamic charges, nonlinear suppression of large magnitude strains, and phase noise analysis. However, low-noise and high-precision oscillating readout circuits for MEMS quartz resonant accelerometers have not been sufficiently investigated, which constrains the potential for further improvement in their stability and resolution.

In this work, the phase noise of piezoelectric quartz resonant accelerometers is systematically analyzed with respect to their unique resonance principle, and a novel oscillating readout circuit topology is proposed to improve the stability and resolution of the accelerometers. A new bandpass and low-noise front-end, capable of delivering higher gain and lower drift, is proposed to overcome the limitations of the traditional front-end in terms of accelerometer performance. An anti-aliasing phase shifter is proposed as a means of achieving additional phase shift compensation and reducing the white-frequency noise in the loop. The nonlinear effect of the MEMS quartz resonator is also considered, and a suitable operating point is set by an amplitude limiter. The proposed MEMS quartz resonant accelerometer has a resolution of 0.26 μg/√Hz and a bias instability of 0.59 μg with a full scale of ±70 g, a scale factor of 54.5 Hz/g, and a bandwidth of 552 Hz.

## Materials and methods

### Design of the quartz-sensitive element

**Fig. 1:**
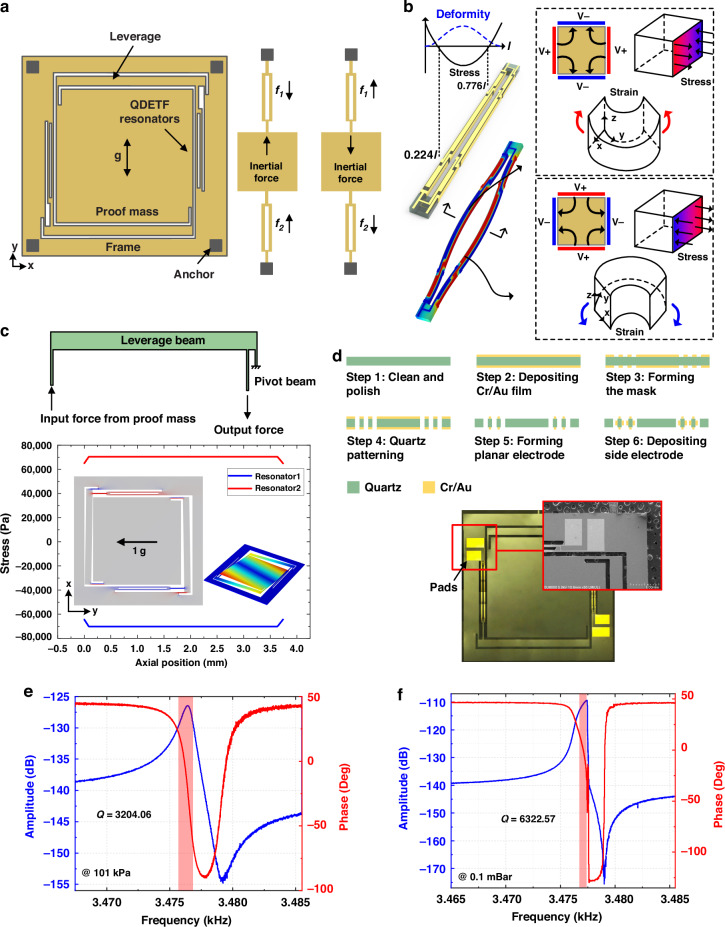
Design, fabrication, and characterization of the accelerometer sensitive element. **a** Schematic of the sensitive element. **b** Principle of piezoelectric quartz resonator and its electrode layout. **c** Schematic of microleverage and finite element simulation of sensitive elements. **d** Fabrication process of the sensitive element and its optical image and SEM image of the electrodes and microleverage. Measured quartz resonator frequency response and Q factor at (**e**) 101 kPa and **f** 0.1 mbar

A resonant accelerometer is a device that measures changes in the intrinsic frequency of sensitive elements under an applied acceleration. Figure 1a shows a schematic of the sensitive element of the MEMS quartz resonant accelerometer proposed in this work, which consists of a proof mass attached to resonators via force-amplifying leverage. Upon the detection of an acceleration signal in the sensitive direction (*y*-axis direction), the proof mass exerts an axial force on the resonator under the influence of inertial force. This force is amplified by the leverage structure, thereby increasing the scale factor of the accelerometer. When the resonator is subjected to axial tensile stress, the resonant frequency increases; conversely, when it is subjected to axial compressive stress, the resonant frequency decreases. To suppress common-mode error interference, the sensitive element proposed in this paper employs a differential resonator structure. When it is subjected to acceleration, the resonance frequency of one of the resonators increases, whereas the other resonance frequency decreases. The difference between the resonance frequency changes of the two resonators is then used as the acceleration measurement value.

Figure 1b presents the operational principle of the quartz resonator, which is a double-ended tuning fork (QDETF) structure with the length direction aligned along the y-axis of the z-cut monocrystalline quartz. When a potential difference exists on the surface of a quartz crystal, internal stresses occur, causing deformation under external constraints, whereas the electric displacement distribution on the surface affects the accumulation of charges that form a current in the appropriate loop. The electrodes are distributed on the width and height surfaces of the QDETF beam, with the symmetry planes comprising electrodes of the same polarity. When one pair of electrodes is at a high potential and the other pair of electrodes is at a low potential, the inverse piezoelectric effect of the quartz crystals causes the cross-section of the resonator beam to generate strain in the *y*-axis and *z*-axis directions. The z-directional strains cancel each other out in the same plane, whereas the y-directional strains result in bending deformation of the resonant beam. To increase the quality factor of the resonator, the electrodes on the QDETF have been carefully designed. The two vibrating beams of the QDETF are considered to have a high stability because the vibrations at the fixed end cancel each other out in the in-plane reversed-phase mode with less coupling to the outside^20,21^. Therefore, we choose this mode as the working mode of the QDETF. The finite element simulation results are shown in Fig. 1b. In this mode, the axial stresses in the vibrating beams are flipped along the length direction at ratios of 0.224 and 0.776. Therefore, three electrodes are designed in each beam, and the electrodes in the same plane are separated at the above ratio positions. To produce a flipped stress distribution at the electrode separation point, it is only necessary to flip the polarity of the neighboring electrodes. We arrange large rectangular electrodes on the surface of the beam and connect them to the side electrodes of the next segment through short wires, which can generate a large electric field and collect abundant charges within a limited cross-section. The results of the electromechanical coupling finite element simulation of the QDETF are shown in Fig. 1b, where the resonator operates in the desired in-plane reversed-phase modes when opposite potentials are applied on the neighboring surfaces of the beam, where the red color represents the high potential and the blue color represents the low potential^20^. The frequency response of the QDETF is tested in a vacuum cavity by adjusting the air pressure. The measured Q factors of the QDETFs are 3204.6 at 101 kPa and 6322.57 at 0.1 mbar, as shown in Fig. 1e and Fig. 1f. In a low-pressure environment, the QDETF exhibits significant nonlinear characteristics, although the damping is smaller, which is related to the stiffness hardening phenomenon of the QDETF. Owing to the reduced damping, the QDETF can easily operate in large amplitude states, which is favorable for the SNR. However, at the same time, it no longer satisfies the Eulerian–Bernoulli beam assumption and the axial stretching of the beam during vibration cannot be disregarded, which induces a change in the resonant frequency of the QDETF. The variation in the QDETF resonance frequency caused by the drive voltage can be expressed as follows:

1 $$\varDelta f=\frac{3Q}{8M{f}_{n}}\frac{{k}_{3}}{{k}_{1}}{K}_{VS}{V}_{drive}(Hz)$$

where *M*, *Q*, and *f*_*n*_ are the mass, *Q* factor, and intrinsic frequency of the resonant beam, respectively, *k*_*3*_/*k*_*1*_ is the ratio of the third stiffness factor to the primary stiffness factor of the QDETF, *K*_*VS*_ is the scale factor from the QDETF drive voltage to the equivalent strain, and *V*_*drive*_ is the drive voltage. When the structural parameters are constant, the larger the drive voltage is, the larger the frequency drift of the QDETF. This drift is independent of the acceleration to which the resonator is sensitive. The measurement accuracy of the accelerometer will be reduced, and methods must be employed to control it. This pattern of behavior is also consistent with the test results presented in Fig. 3e.

A level of air damping can improve the high-frequency response of the accelerometer, particularly by suppressing the limitation of the operating bandwidth of the accelerometer caused by high-frequency vibration disturbances. Moreover, the proximity of the package pressure to the ambient pressure helps reduce the leakage rate of the package and improves the long-term stability of the accelerometer. Therefore, in this work, an atmospheric pressure nitrogen encapsulation method is used to achieve acceptable resonator Q-factors and long-term stable accelerometer performance while reducing production and maintenance costs.

To provide the sensitivity of the resonant accelerometer, we amplify the inertial force sensitive to the proof mass by using a microleverage structure, as shown in Fig. 1c. This structure consists of an input beam, a leverage beam, an output beam, and a pivot beam, which are installed the edge of the frame for compact device dimensions. According to the equilibrium, physical, and deformation coordination equations, the amplification factor *K* of the microleverage can be calculated as follows:

2 $$K=\frac{{F}_{out}}{{F}_{in}}=\frac{({L}_{in}+{L}_{out})\cdot {L}_{out}+\frac{{I}_{oz}{l}_{p}+{I}_{pz}{l}_{o}}{{A}_{p}{l}_{p}}}{{{L}_{out}}^{2}+\frac{({I}_{oz}{l}_{p}+{I}_{pz}{l}_{o})\cdot ({A}_{o}{l}_{p}+{A}_{p}{l}_{o})}{{A}_{o}{A}_{p}{l}_{o}{l}_{p}}}$$

where *A*_*p*_ *= h*_*p*_*b*_*p*_, *A*_*o*_ *= h*_*o*_*b*_*o*_, *I*_*pz*_ *= b*_*p*_*h*_*p*_^3^*/12*, and *I*_*oz*_ *= b*_*o*_*h*_*o*_^3^*/12*, *L*_*in*_ is the length of the input force arm, *L*_*out*_ is the length of the output force arm, *b*_*p*_, *h*_*p*_, and *l*_*p*_ are the thickness, width, and length of the pivot beam, respectively, and *b*_*o*_, *h*_*o*_, and *l*_*o*_ are the thickness, width, and length of the output beam, respectively. Figure 1c shows the finite element simulation results of the sensitive element under a unit acceleration load. The proof mass pushes the microleverage to exert an axial force on the resonators when it is subjected to inertial force; one of the resonators is subjected to a compressive stress of −70,500 Pa, denoted in blue, and the other resonator is subjected to a tensile stress of 70,500 Pa, denoted in red, which exhibits good differential performance. The stress at the input end of the microleverage is 4.2 kPa, and the stress at the output end of the microleverage is 157.2 kPa, which is substituted into (2) to obtain a microleverage amplification factor of 38. The first-order mode of the sensitive element in the acceleration-sensitive direction is a torsional vibration of the proof mass in the coupled *x*- and *y*-axes, with an eigenfrequency of 670.2 Hz, which ensures a sufficient bandwidth.

The accelerometer proposed in this work is fabricated on a 100 μm thick z-cut quartz wafer via the MEMS wet etching process, as shown in Fig. 1d. The fabrication process can be completed in six simple steps, with low cost, good consistency, and high-quality mass production capability. First, the quartz wafer was cleaned and polished. Second, a chromium/gold (Cr/Au) film is deposited on its upper and lower surfaces. Third, the Cr/Au film is patterned as a mask layer for wet etching. Fourth, planar electrodes are patterned on the Cr/Au film after sensitive element structure etching is completed. Last, the side electrodes for the QDETF are deposited. The sensitive element fabricated in quartz is shown in Fig. 1d and has dimensions of 15 mm × 15 mm. The pads are designed on the frame to connect to the external electronics.

### Phase noise of the piezoelectric oscillation system

The oscillating readout circuit and the quartz resonator form a piezoelectric oscillation system that maintains the resonant state after power-up and tracks the change in resonant frequency within the full range, allowing the oscillation frequency to be continuously read out to measure the input acceleration signal. The purity and stability of the oscillation frequency are essential for extracting the intrinsic frequency of the resonator. Existing oscillating read-out circuits utilize a dual inverter feedback topology owing to its compactness and low cost^15,22,23^. When the MEMS quartz resonator vibrates, the charge-enriched electrodes are detected and provide feedback to the drive electrodes through the dual-inverter oscillating readout circuit (DIC), which starts up when the amplitude and phase satisfy the Barkhausen stability criterion. The MEMS quartz resonator eventually achieves a steady state owing to mechanical or semiconductor nonlinearities^24^.

**Fig. 2:**
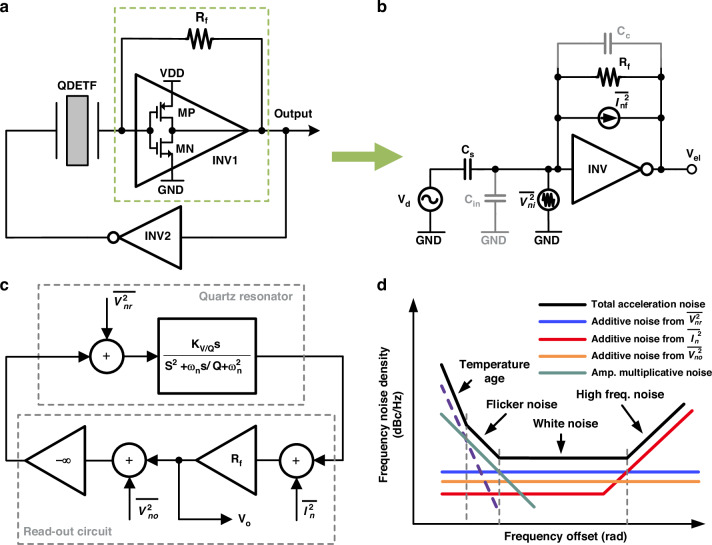
Typical piezoelectric quartz oscillation system and its phase noise modulation model. **a** Schematic of the dual inverter readout circuit. **b** Transimpedance inverter front-end with noise sources. **c** Block diagram of the piezoelectric quartz oscillation system and **d** its output frequency noise spectrum

A simplified diagram of the DIC is shown in Fig. 2a. INV 1 across the resistor is used to detect the motional charge and provide gain to satisfy the amplitude condition. INV 2 feeds the detected phase back to the drive electrode to compensate for the phase drift. A schematic of the transimpedance inverter front-end is shown in Fig. 2b. The inverter adopts a single-sided conduction structure controlled by an external signal formed by the push‒pull of the NMOS and PMOS and provides a feedback loop through the transimpedance at the input and output terminals. The transfer function of the transimpedance inverter front-end can be expressed as

3 $$H(s)=\frac{{V}_{el}}{{V}_{d}}=\frac{{R}_{f}{C}_{c}{A}_{o}{\omega }_{p1}}{{R}_{f}{C}_{in}{C}_{c}{s}^{2}+{R}_{f}{C}_{c}^{2}{A}_{o}{\omega }_{p1}s++{C}_{c}{A}_{o}{\omega }_{p1}}\cdot {C}_{s}s$$

where *C*_*c*_ is the parasitic from the transimpedance *R*_*f*_, *A*_*o*_ and *ω*_*p1*_ are the open-loop gain and first main pole of the inverter, respectively, *C*_*in*_ is the input parasitic, relating to the electromechanical attachment, and *C*_*s*_ is the equivalent capacitance of the mechanical structure.

The gain and bandwidth can be simplified to *R*_*f*_ and 1/*R*_*f*_ *C*_*c*_, respectively, when the transimpedance inverter front-end operates in the linear region, with a severe tradeoff. The input-referred current noise can be expressed as

4 $${\bar{I}}_{n}^{2}(s)=\frac{4kT}{{R}_{f}}+{\bar{V}}_{n}^{2}{\left[\frac{1+{R}_{f}({C}_{in}+{C}_{c})s}{{R}_{f}}\right]}^{2}$$

From above, there are significant constraints on the input-referred current noise, gain, bandwidth, and stability of the transimpedance inverter front-end that limit the improvement in the resolution of MEMS quartz resonant accelerometers.

The frequency stability of the oscillation system determines the bias instability and resolution of the accelerometer. Phase noise is the random phase jitter of the oscillation signal and is often used to evaluate oscillator stability. The overall block diagram of the piezoelectric oscillation system is shown in Fig. 2c. The quartz resonator operates at a high Q factor as an acceleration-sensitive frequency selection network and is equated to a second-order system. The DIC can be abstracted as a gain and phase detector. The output frequency noise spectrum of a typical MEMS quartz resonant accelerometer, including the Brownian thermal noise of the quartz resonator and electronic noise introduced by the oscillating readout circuit, is shown in Fig. 2d. The low-frequency region is attributed mainly to the temperature drift and aging of the resonator, which can be effectively suppressed by compensation techniques. The flicker noise comes from the nonlinear effect of the resonator and can be expressed as

5 $${S}_{1/f}^{2}=\frac{3}{4}\frac{{k}_{3}}{{k}_{1}}{\varepsilon }_{0}\frac{{\bar{A}}_{n}^{2}}{{R}_{f}{K}_{V/Q}}(dBc/Hz)$$

where *ε*_*0*_ and ${\bar{A}}_{n}^{2}$ are the strain and amplitude noise density of the quartz resonator, respectively, *R*_*f*_ is the gain of the front-end, and *K*_*V/Q*_ is the conversion factor from the quartz resonator drive voltage to the motional charge determined by mechanical parameters. The white noise defines the resolution of the MEMS quartz resonant accelerometer, which consists of two uncorrelated components: MEMS quartz resonators’ Brownian thermal noise ${\bar{V}}_{nr}^{2}$ and electronic noise, mainly from the front-end’s noise ${\bar{I}}_{n}^{2}$ and the inverter’s voltage noise ${\bar{V}}_{no}^{2}$. Based on Fig. 2c, ${\bar{V}}_{nr}^{2}$, ${\bar{I}}_{n}^{2}$ and ${\bar{V}}_{no}^{2}$ induced additive noises can be expressed as

6 $${S}_{f1}^{2}=\frac{4{R}_{f}^{2}{\bar{V}}_{nr}^{2}}{{Q}^{2}{R}_{eq}^{2}{K}_{V/Q}^{2}{\varepsilon }_{0}^{2}}(dBc/Hz)$$

7 $${S}_{f2}^{2}=\frac{4{R}_{f}^{2}{\bar{I}}_{n}^{2}}{{Q}^{2}{K}_{V/Q}^{2}{\varepsilon }_{0}^{2}}(dBc/Hz)$$

8 $${S}_{f3}^{2}=\frac{4{R}_{f}^{2}{\bar{V}}_{no}^{2}}{{Q}^{2}{R}_{eq}^{2}{K}_{V/Q}^{2}{\varepsilon }_{0}^{2}}(dBc/Hz)$$

where *R*_*eq*_ is the equivalent resistance of the resonator.

Thus, the resolution *R* of the accelerometer is given by

9 $$R=\frac{{f}_{n}\sqrt{{S}_{f1}^{2}+{S}_{f2}^{2}+{S}_{f3}^{2}}}{SF}(g/\sqrt{Hz})$$

where *SF* is the scale factor of the accelerometer. From Fig. 2d and (9), to improve the resolution of the MEMS quartz resonant accelerometer, it is possible to (i) increase the *Q* factor, *ε*_*0*_ or *SF*, (ii) reduce the *f*_*n*_, (iii) optimize the mechanical parameters of the quartz resonator to improve its *K*_*V/Q*_ and (iv) minimize electronic noise in circuits. White noise optimization can directly improve the resolution of accelerometers without changing their mechanical properties and simultaneously has the most universal value for other piezoelectric devices.

### Proposed low-noise oscillating read-out circuit

#### System overview

**Fig. 3:**
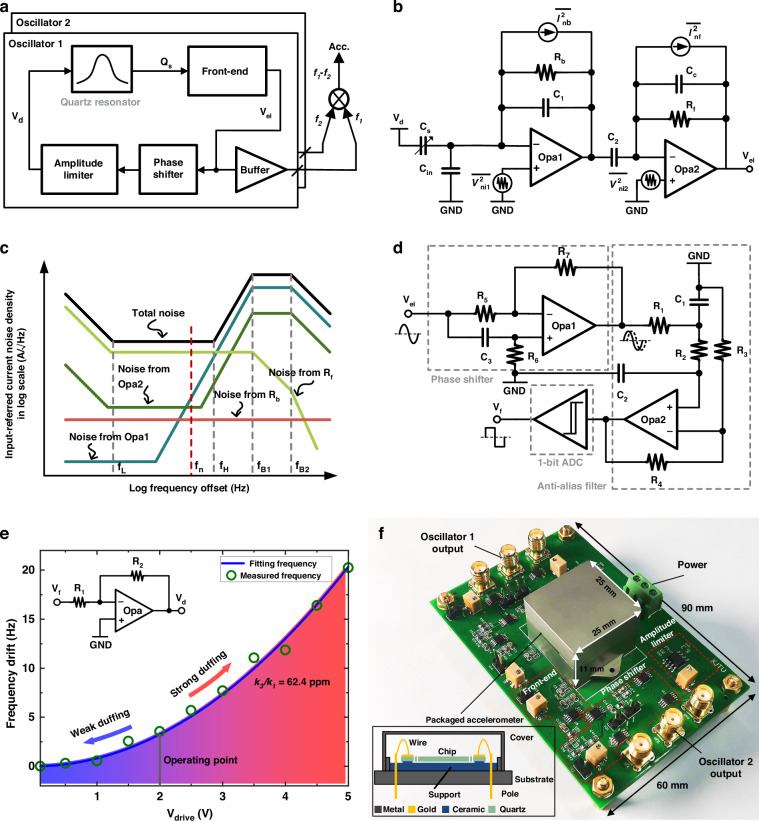
Proposed novel low-noise oscillating system and its implementation. **a** System block diagram of the proposed MEMS quartz resonant accelerometer. **b** Opa-based low-noise bandpass front-end. **c** Input-referred current noise density of the proposed bandpass front-end. The spectrum before *f*_*H*_ is dominated by the parameters of the feedback circuit and the roll-off frequency is dominated by the Opa poles, which can be used to limit the loop bandwidth and reduce the system noise. **d** Schematic of the Anti-aliasing phase shifter. **e** Quartz resonator frequency drift under variable voltage. **f** Photograph of a packaged MEMS quartz resonant accelerometer with an oscillating PCB

A novel low-noise oscillating readout circuit (LNC) is proposed and shown in Fig. 3a. The operational amplifier (Opa)-based front-end detects the motional charge of the operating mode of the quartz resonator and outputs a voltage proportional to its mode velocity. The phase shifter satisfies the oscillation condition. The amplitude limiter feeds the oscillation to the quartz resonator and sets the operating point to minimize the modulation of flicker noise. A buffer followed by the front-end provides the load-carrying capability. The outputs of the two oscillators are frequency-differenced by a multiplier to enable acceleration measurements.

From the phase noise decomposition model, the white noise introduced at the front-end is the key to limiting the resolution of MEMS quartz resonant accelerometers.

#### Low-noise bandpass front-end

Figure 3b shows the schematic of the proposed bandpass front-end, which consists of two stages. The first stage can be considered an integrator that outputs a voltage proportional to the strain of the QDETF by integrating the charge generated by the surface of the QDETF during vibration through *C*_*1*_. *R*_*b*_ is used to provide DC feedback to the Opa, and the values of *R*_*b*_ and C_1_ are chosen to determine the low-pass cutoff frequency *f*_*L*_. The second stage acts as a differentiator to offset the phase shift introduced by the first stage to meet the phase conditions of the oscillation. *C*_*c*_ is used to compensate for the stability and determine the high-pass cutoff frequency *f*_*H*_. The topology of the integrator in series with the differentiator creates a bandpass frequency response that provides a gain of *R*_*f*_ *C*_*2*_/*C*_*1*_ when the oscillation frequency lies within the passband of the front-end.

The transfer function of the bandpass front-end can be expressed as

10 $$H(s)=\frac{{R}_{b}{R}_{f}{C}_{2}{C}_{s}{s}^{2}}{({R}_{b}{C}_{1}s+1)({R}_{f}{C}_{c}s+1)}$$

An important observation from the above discussion is that the gain and bandwidth of the bandpass front-end can be designed independently, which eliminates the tradeoff in the performance of the traditional transimpedance inverter front-end. The oscillation frequency *f*_*n*_ should satisfy the following

11 $$\frac{10}{\pi {R}_{b}{C}_{1}}\, <\, {f}_{n} < \frac{1}{40\pi {R}_{f}{C}_{c}}$$

Large parasitics tend to destabilize the stability of the front-end and limit its bandwidth. The open loop transfer function of the bandpass front-end can be written as

12 $$A(s)=\frac{{A}_{o}{C}_{s}s({R}_{b}{C}_{1}s+1)}{[{R}_{b}({C}_{1}+{C}_{in})s+1](1+s/{\omega }_{p1})(1+s/{\omega }_{p2})}$$

where *A*_*o*_ is the DC gain of Opa and *ω*_*p1*_ and *ω*_*p2*_ are its first pole and second pole, respectively. From (12), the introduced pole and zero of *C*_*in*_ are compensated as long as *R*_*b*_*C*_*1*_*ω*_*p1*_ > 1 is satisfied, thereby ensuring stability.

As shown in Fig. 3b, the noise at the bandpass front-end can be modeled by four uncorrelated noise sources, including the voltage noises from both Opa and the current noises from the resistance with densities ${\bar{V}}_{ni1}^{2}$, ${\bar{V}}_{ni2}^{2}$, ${\bar{I}}_{nb}^{2}$ and ${\bar{I}}_{nf}^{2}$. Its input-referred current noise ${\bar{I}}_{ni}^{2}$ can be computed as

13 $$\begin{array}{c}{\bar{I}}_{ni}^{2}={\bar{I}}_{nb}^{2}+{\bar{I}}_{nf}^{2}{(\frac{{R}_{b}{C}_{1}s+1}{{R}_{b}{C}_{2}s})}^{2}+{\bar{V}}_{ni1}^{2}{[\frac{1}{{R}_{b}}+s({C}_{1}+{C}_{in})]}^{2}\\ +{\bar{V}}_{ni2}^{2}{\left(\frac{{R}_{f}({C}_{c}+{C}_{2})s+1}{{R}_{f}{C}_{2}s}({C}_{1}s+\frac{1}{{R}_{b}})\right)}^{2}({A}^{2}/Hz)\end{array}$$

Figure 3c shows the total input-referred current noise spectrum. Within the passband of the front-end, the ${\bar{I}}_{ni}^{2}$ is dominated by ${\bar{I}}_{nb}^{2}$ and ${\bar{I}}_{nf}^{2}$, and *R*_*b*_ is much larger than *R*_*f*_; therefore, the differentiator current noise limits the noise level in this band. The input reference current noise decreases by 20 dB/dec when it is greater than *f*_*H*_, which is modulated into the quantization noise in the oscillator. *f*_*B1*_ and *f*_*B2*_ are determined by the bandwidth of the Opa. To minimize the effect of the oscillating readout circuit on the stability of the oscillator, the following conditions are recommended:

14 $${R}_{b}{C}_{1} \,>\, {R}_{f}{C}_{2}$$

15 $${\bar{V}}_{ni1}^{2}{(2\pi {f}_{n}{C}_{1})}^{2}\, <\, {\bar{I}}_{nf}^{2}$$

16 $${\bar{V}}_{ni2}^{2}{(2\pi {f}_{n}{C}_{2})}^{2} \,<\, {\bar{I}}_{nf}^{2}$$

Compared with the traditional transimpedance inverter front-end, the proposed bandpass front-end has several advantages, as follows:

(i) the individually designable gain and bandwidth eliminate the tradeoff between them, making it possible to achieve high gain and large bandwidth simultaneously;
(ii) the noise of the Opa is suppressed by the integrator, and the very low pole of the integrator reduces the current noise introduced by *R*_*b*_;
(iii) the poles introduced by *C*_*in*_ can be easily compensated by a large *R*_*b*_ without stability risks.

Based on (13), larger *C*_*2*_/*C*_*1*_ and *R*_*f*_ values enhance the gain and reduce the input-reference current noise of the novel front-end. Considering the mechanical parameters of our accelerometer, *C*_*1*_, *C*_*2*_, and *R*_*f*_ are 10 pF, 15 nF, and 9 kΩ, respectively. The resistances *R*_*b*_ and *C*_*c*_ are 5 GΩ and 10 pF, respectively, to ensure the DC gain of the first-stage Opa and to compensate for the stability of the second-stage Opa. The total gain is thus 14.5 M with a 0.04° phase drift at the oscillation frequency. The simulated value of the input-referred current noise is 27.6 fA/√Hz with a precision Opa.

#### Anti-aliasing phase shifter

To satisfy the phase conditions of oscillation, the mode phase of the QDETF must be detected, and a smaller error is required to ensure the stability of the oscillator. The analog-to-digital converter (ADC) is a convenient device for estimating the phase information of a signal. However, a high SNR is often required to reduce the risk of false triggering, which limits the application of low-amplitude QDETF oscillators. In addition, the aliasing noise introduced by ADC undersampling can significantly contaminate the white noise of the oscillator and restrict the accelerometer resolution. Therefore, this work proposes an anti-aliasing phase shifter that introduces negligible aliasing noise and compensates for the phase error caused by the oscillating readout circuit to realize high-stability oscillation.

The proposed anti-aliasing phase shifter consists of an analog phase shifter, an anti-aliasing filter, and an ADC, as shown in Fig. 3d. The phase shifter is used to compensate for the phase drift introduced by the prestige for better oscillation accuracy. Owing to the demanding precise phase shift requirements of electronic components and the subsequent increase in manufacturing costs, this paper employs a 50 kΩ precision variable resistor *R*_*6*_ in conjunction with a 45 nF fixed capacitor *C*_*3*_ to form an overshooting phase compensator. This compensator continuously adjusts the compensated phase through the value of *R*_*6*_ to achieve the optimum Q factor of the oscillator. Since the oscillator noise is distributed over the entire bandwidth, to minimize the introduction of excessive aliasing noise during the phase extraction of the ADC, a second-order lowpass filter is designed in front of the ADC to limit the loop noise bandwidth. The cutoff frequency is 20 times the resonant frequency of the QDETF, i.e., 700 kHz, which effectively reduces aliasing noise, ensures the stability and resolution of the accelerometer, and is achieved without affecting the phase shift. A 1-bit ADC with a carefully designed hysteresis range outputs a square signal, which drives the quartz resonator to ensure fast and robust oscillation start-up.

#### Amplitude limiter

The mode strain of the QDETF must be limited to a small value (within 2 V in this work) to avoid strong nonlinearities that modulate the noise from the amplitude domain to the phase domain, which can degrade the stability of the accelerometer. The frequency response of the QDETF was measured at different drive voltages to identify the resonant frequency. The variation in the resonant frequency of the QDETF with the drive voltage is plotted in Fig. 3e. The nonlinear effect of the QDETF gradually enhances with increasing drive voltage. Fitting the measured values by (1) yields a *k*_*3*_/*k*_*1*_ of 62.4 ppm for the QDETF proposed in this work, which is much lower than those of previous comparable works^17,25^, as well as silicon resonant accelerometers^26,27^. However, the amplitude of the QDETF still needs to be limited for better stability.

Automatic amplitude control techniques are often used to set the mode amplitude of MEMS resonators. However, they tend to introduce excessive flicker noise, which destabilizes the long-term stability of the accelerometer^28–31^. In this work, a simple yet effective amplitude-limiting strategy is employed, which is based on the digital signal output from the ADC. This strategy limits the amplitude of the drive signal through an amplifier with a gain of less than one, thus controlling the QDETF to operate in its weakly nonlinear region. Furthermore, the power consumption of the system is reduced without the introduction of excessive noise. Since the carrier power is equally important to the SNR of the accelerometer, the constraints between them are balanced. The experiments verify the effectiveness of the strategy.

## Results and discussion

The oscillating readout circuit is fabricated on a printed circuit board (PCB) with dimensions of 90 mm × 60 mm. The metal case is soldered to the PCB by poles. The packaged MEMS sensitive element with an oscillating readout circuit PCB is shown in Fig. 3f. To further minimize the interference of thermal stress, the sensitive element is isolated from the substrate by ceramic support, which provides active space for the resonator and has a thermal expansion coefficient close to that of quartz. The pads on the QDETFs are bonded by a gold wire, which results in poles running through the substrate to form an oscillator with external electronics. A cover is welded to the substrate to create a sealed working environment for the sensitive element. The overall power consumption of the accelerometer is 80 mW with a 5 V supply. The oscillation readout circuit is arranged outside the package case for optimal heat dissipation, which minimizes its impact on the temperature performance of the accelerometer. Furthermore, the thermal contribution of the circuit is effectively suppressed by the differential QDETF.

### Front-end test results

**Fig. 4:**
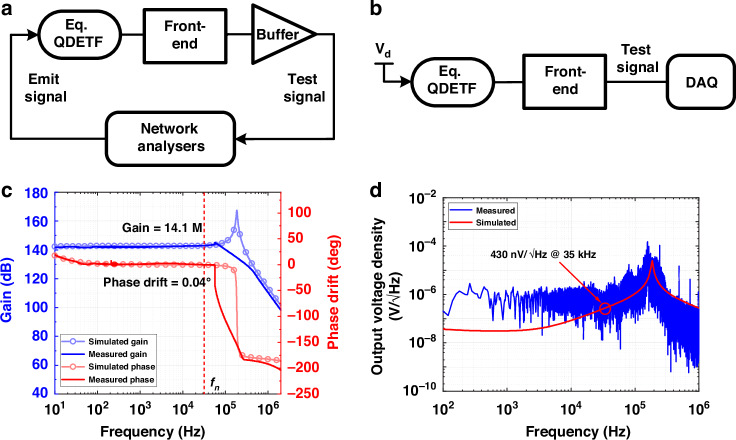
Front-end experimental setup and test results. Experimental setup for (**a**) frequency response and (**b**) noise performance. Measured (**c**) frequency response and (**d**) output voltage noise density of the proposed front-end via simulation

Figure 4c shows the measured frequency response of the proposed front-end in this work. The gain and phase drift are 14.1 M and 0.04° at the oscillation frequency, respectively. A carefully chosen Opa is used to build the front-end. Owing to its limited bandwidth, the results show a sharp degradation of the response above 60 kHz, where the frequency is much higher than the oscillation frequency. This phenomenon can be attributed to the design of the front-end differentiator, which places the main poles near the Opa bandwidth. When the two poles coincide, a significant degradation in the frequency response results. Therefore, the phase drift can be disregarded, which is equivalent to a high-order low-pass filter that can effectively suppress high-frequency noise and reduce the modulated white noise from the accelerometer.

The measured output voltage noise spectrum of the proposed front-end is shown in Fig. 4d. The voltage noise is flat in the passband and increases above a specific frequency. This finding is consistent with the analysis above. The output voltage noise at 35 kHz is 430 nV/√Hz, which corresponds to a low input-referred current noise of 30.5 fA/√Hz. The reason for the test values being slightly higher than the simulated values is probably due to the presence of unexpected parasitics at the actual circuit connections.

### Noise test results

**Fig. 5:**
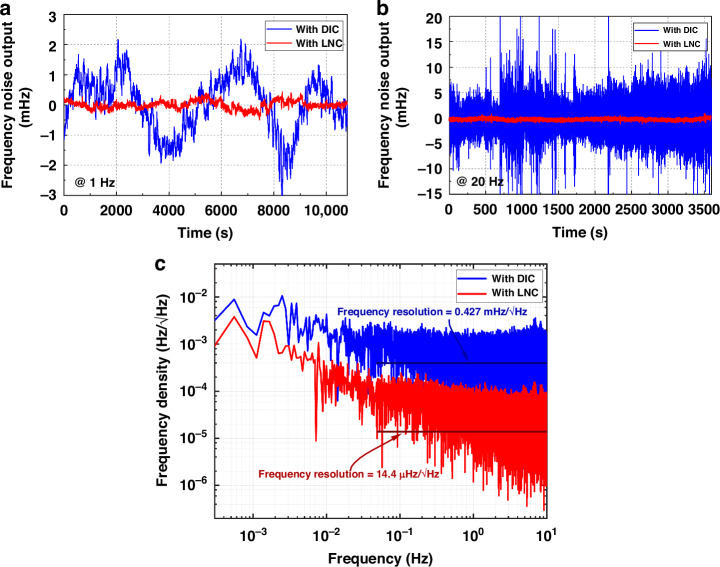
Noise test results of the oscillating readout circuit. 0 g acceleration output at room temperature for (**a**) 3 h at 1 Hz sample rate and (**b**) 1 h at 20 Hz sample rate. **c** Measured frequency noise density

The accelerometer is fixed to the index head, the frequency of the two oscillator output signals is measured by a commercial frequency meter, the Keysight 53230A and the difference is calculated in the host computer to recognize the magnitude of the input acceleration. Accelerometer output drift with temperature is unavoidable due to the different coefficients of thermal expansion of the materials; hence, temperature compensation is necessary. The quartz resonant accelerometer proposed in this work exhibits excellent performance with only simple linear fitting compensation. Noise tests are performed with the same accelerometer coupled with the traditional DIC and the LNC proposed in this work. Figure 5a shows the differential frequency noise output of the accelerometer with each of the two circuits for 3 h at room temperature at a sampling rate of 1 Hz. The measured frequency output with the LNC varies within 0.9 mHz over 3 h, with a standard deviation of 0.1 mHz. This value is 5.5 times greater than that with DIC. Figure 5b shows the change in the accelerometer noise output over 1 h using both circuits at a sample rate of 20 Hz. The standard deviation decreases from 2.6 mHz for the DIC to 0.3 mHz for the LNC.

Figure 5c shows the frequency noise density of the accelerometer at 0 g. The LNC has a noise floor of 14 μHz/√Hz, which is significantly lower than the noise floor of the DIC of 0.427 mHz/√Hz. The detailed performance comparisons in Table 1 show that the accelerometer with the LNC outperforms the accelerometer with the DIC by one order of magnitude.

**Table 1:** Performance comparison of the two circuit topologies

Circuit	Maximum variation (mHz)		Deviation (mHz)		Frequency resolution (mHz/√Hz)
	@ 1 Hz	@20 Hz	@ 1 Hz	@20 Hz	/
DIC	5	35	0.85	2.6	0.427
LNC	0.9	2	0.1	0.3	0.014

### Accelerometer test results

**Fig. 6:**
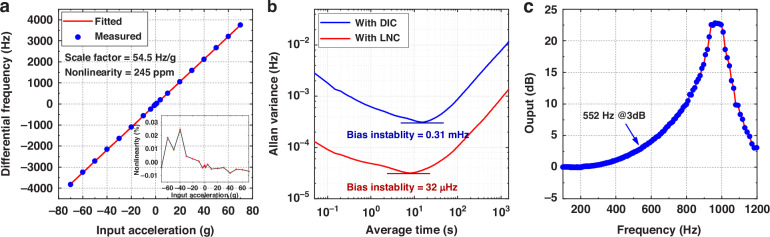
Accelerometer performance test results. Measured (**a**) Full scale with scale factor, (**b**) Allan variance, and (**c**) Bandwidth

The accelerometer was attached to a centrifuge to measure the variations in the output for different acceleration inputs. Figure 6a shows the full-scale acceleration test results under ±70 g input. The measured scale factor is 54.5 Hz/g, with a maximum nonlinearity of 245 ppm. Figure 6b shows the measured Allan variance of the MEMS quartz resonant accelerometer with the LNC, with a bias instability of 32 μHz; this value is reduced from 0.31 mHz with the DIC. The acceleration resolution and bias instability are 0.26 μg/√Hz and 0.59 μg, respectively. The accelerometer was subsequently fixed to the vibration generator, the vibration signal was input, and the accelerometer output was recorded to obtain its frequency response, as shown in Fig. 6c. As the frequency of the vibration signal approached the intrinsic frequency of the accelerometer, a resonance peak appeared in the output response, which yielded an accelerometer bandwidth of 552 Hz.

Table 2 summarizes the measured performance of the MEMS quartz resonant accelerometer proposed in this work and compares it with previous art, including both quartz and silicon MEMS resonant accelerometers. To compare the noise performance of the oscillators themselves, the frequency resolution and frequency instability are calculated for each reference. The resolution and instability of the quartz oscillator are better than those of the silicon oscillator. The oscillator in this paper achieves the best performance. The work in 6 reached a lower acceleration resolution owing to a larger scale factor but had poor bias instability and a smaller full scale. The accelerometer proposed in this work results in better overall performance.

**Table 2:** Comparison of the previous MEMS accelerometer with an oscillating readout circuit

Reference	6	30	31	29	25	15	This work
Mechanism	Electrostatic resonant	Electrostatic resonant	Electrostatic resonant	Piezoelectric resonant	Piezoelectric resonant	Piezoelectric resonant	**Piezoelectric resonant**
Full scale (g)	±25	±0.32	±25	±50	±50	±10	**±70**
Nonlinearity (ppm)	262	–	1000	–	–	200	**245**
Scale factor (Hz/g)	45.8	776.75	1110	150	60	345	**54.5**
Bandwidth (Hz)	–	–	–	500	–	45	**552** @3 dB
Resolution (μg/√Hz)	5.8	7.04	0.7	0.16	3	1.9	**0.26**
Bias instability (μg)	5.7	13.15	0.123	0.64	1	0.7	**0.59**
Frequency resolution (mHz/√Hz)	0.26	5.47	0.78	0.024	0.18	0.66	**0.014**
Frequency instability (mHz)	0.27	9.98	0.14	0.096	0.06	0.25	**0.032**

## Conclusion

A differential MEMS quartz resonant accelerometer with a novel oscillating readout circuit is implemented. The phase noise modulation mechanism of MEMS quartz resonant accelerometers is analyzed in detail, and the front-end performance is demonstrated to be a key factor in determining the stability and resolution of accelerometers. A novel low-noise bandpass front-end is proposed to eliminate the tradeoff among gain, bandwidth, and noise in conventional front-ends. The test results demonstrate that this topology provides a gain of 14.1 M and a phase drift of 0.04° at the oscillation frequency, accompanied by input-referred current noise as low as 30.5 fA/√Hz. Benefiting from excellent front-end performance, carefully designed phase compensation, and the resonator operating point, the MEMS quartz resonant accelerometer in this work has achieved a frequency resolution of 14 μHz/√Hz and frequency instability of 32 μHz, which corresponds to a resolution of 0.26 μg/√Hz and a bias instability of acceleration of 0.59 μg, with a scale factor of 54.5 Hz/g, a bandwidth of 552 Hz, and a full scale of ±70 g, and state-of-the-art performance.
